# IL-22: An Underestimated Player in Natural Resistance to Tuberculosis?

**DOI:** 10.3389/fimmu.2018.02209

**Published:** 2018-09-25

**Authors:** Katharina Ronacher, Roma Sinha, Michelle Cestari

**Affiliations:** ^1^Division of Molecular Biology and Human Genetics, Faculty of Medicine and Health Sciences, SAMRC Centre for Tuberculosis Research, DST-NRF Centre of Excellence for Biomedical Tuberculosis Research, Stellenbosch University, Cape Town, South Africa; ^2^Infection, Immunity and Metabolism Group, Translational Research Institute, Mater Research Institute and The University of Queensland, Brisbane, QLD, Australia

**Keywords:** tuberculosis, *Mycobacterium tuberculosis*, interleukin-22, IL-22R1, T lymphocytes, respiratory infections

## Abstract

Approximately 10% of individuals latently infected with *Mycobacterium tuberculosis* (Mtb) develop active tuberculosis (TB) during their lifetime. Although it is well recognized that T-helper 1 immune responses are crucial for containing latent TB infection, the full array of host factors conferring protective immunity from TB progression are not completely understood. IL-22 is produced by cells of the innate and adaptive immune system including innate lymphoid cells, and natural killer cells as well as T lymphocytes (Th1, Th17, and Th22) and binds to its cognate receptor, the IL-22R1, which is expressed on non-hematopoietic cells such as lung epithelial cells. However, recent studies suggest that Mtb induces expression of the IL-22R1 on infected macrophages and multiple studies have indicated a protective role of IL-22 in respiratory tract infections. Reduced concentrations of circulating IL-22 in active TB compared to latent TB and decreased percentages of Mtb-specific IL-22 producing T cells in TB patients compared to controls designate this cytokine as a key player in TB immunology. More recently, it has been shown that in type 2 diabetes (T2D) and TB co-morbidity serum IL-22 concentrations are further reduced compared to TB patients without co-morbidities. However, whether a causative link between low IL-22 and increased susceptibility to TB and disease severity of TB exists remains to be established. This review summarizes the contribution of IL-22, a potentially under-appreciated key player in natural resistance to TB, at the interface between the immune response to Mtb and the lung epithelium.

## Introduction

A quarter of the human population is infected with *M. tuberculosis* (Mtb) (1) of which ~10% will develop the active and contagious form of tuberculosis (TB) during their lifetime (2). Various intrinsic and extrinsic factors determine the natural course of mycobacterial infection, and resistance vs. susceptibility to disease progression. These factors include host genetic susceptibility (3), virulence of the infecting strain (4) and presence of acquired immune deficiencies such as HIV infection and type 2 diabetes (T2D) (5). The role of IL-22 during the host defense against Mtb is poorly understood. The subsequent sections highlight our current knowledge of the protective function of IL-22 during respiratory tract infections, including TB.

## Source and targets of IL-22

IL-22 is produced by cells of the innate as well as the adaptive immune system including tissue resident innate lymphoid cells (ILCs), NK cells, macrophages, NKT cells, activated Th1, Th17, and Th22 cells as well as Tc-cell subsets and γδ T cells (6). Alveolar macrophages from both humans and mice are also able to produce and release IL-22 (7). In mice, antigen-specific IL-22 production is driven by Th1 and Th17 cells, but only a small subset of Th17 cells produce IL-22 in humans. In contrast to mice, humans have a distinct subset of T helper cells, called Th22 cells, which produce IL-22 and TNFα. Unlike Th1 and Th17 subsets, human Th22 cells, which were initially characterized in skin neither produce IL-17 nor IFNγ (8). Apart from secreting IL-22, Th22 cells can also express granzymes, IL-13 and increased levels of Tbet showing a remarkable plasticity to skew the immune response toward pro- or anti-inflammatory depending on the Th1 or Th2 stimulus *in vitro* (9).

IL-22 binds to its heterodimeric receptor complex consisting of the IL-22R1 and the IL-10R2 to activate the JAK-STAT signaling pathways (10). The IL-22R is present on epithelial cells of the lung, gut and skin, the liver, pancreas, and kidneys. It is not expressed on hematopoietic cells, neither in resting/naïve nor activated macrophages, T or B cells, nor the human monocyte THP-1 cell-line (11, 12). However, three independent studies reported that Mtb induces expression of the IL-22R1 in infected macrophages (13–15). The significance of this is discussed in the next section.

A T cell-derived soluble IL-22 binding protein (IL-22BP), which shares sequence homology with the extracellular domain of the membrane bound IL-22R1, acts as endogenous inhibitor of IL-22 by preventing its binding to the IL-22R1. Activation of the IL-22 signaling pathway in epithelial cells results in epithelial tissue proliferation, regeneration, and healing, therefore this cytokine plays an important role in protection from infection-induced tissue damage at mucosal surfaces (10). IL-22 induces expression of the chemokines CXCL1 and CXCL5 in bronchial epithelia in a *Klebsiella pneumonia*e infection model (16), but reduces CXCL8, a neutrophil attracting chemokine, in A459 human lung carcinoma cells (17). Most importantly, IL-22 stimulates the production of antimicrobial peptides such as β-defensins, the S100 family of peptides, Reg3β and γ, lipocalin-2, calprotectin and calgranulin A in various cell types (18–21), thereby controlling bacterial growth and reducing the risk of secondary bacterial infections after viral injury (22).

## IL-22 as immune-modulator to inhibit mycobacterial growth

In addition to the well-described effect of IL-22 on epithelial cells the recent reports that Mtb induces expression of the IL-22R1 on macrophages, the primary host immune cells targeted by mycobacteria, is particularly intriguing. Treerat and colleagues report IL-22R1 positive macrophages by immunohistochemistry in granulomas of HN878 infected mice, but whether this positive signal is due to HN878 induced IL-22R1 expression on macrophages or through ingestion of IL-22R1 positive epithelial cell debris by the lung macrophages remains to be confirmed (15). Two previous studies report a modest induction of IL-22R1 expression on macrophages after stimulation with Mtb H37Rv and Erdman by flow cytometry (13, 14). Upregulation of the IL-22R1 in infected macrophages may be a host-mechanism to combat the infection, as there is growing evidence that IL-22 can modulate mycobacterial growth within macrophages.

In initial experiments Dhiman et al. observed that Mtb-infected human monocytes induce production of IL-22 by co-cultured autologous NK cells in a IL-15 and IL-23 dependent manner. This NK mediated IL-22 production resulted in reduction of intra-macrophagic bacteria and was reversed through neutralization of IL-22 suggesting that the mycobacterial growth inhibition is at least in part attributable to IL-22 (13). In subsequent experiments by the same group exogenous addition of recombinant IL-22 (rIL-22) to infected macrophages promoted phagolysosomal fusion and reduced bacterial burden (23). The anti-mycobacterial activity of IL-22 was mediated through increased expression of the anti-microbial peptide calgranulin A and siRNA knock down of calgranulin A abrogated the IL-22 dependent mycobacterial containment in monocyte derived macrophages (23). An additional mechanism by which IL-22 may contribute to reduction in mycobacterial burden is the observed increased TNFα production by Mtb-infected bone marrow-derived macrophages when pre-treated with rIL-22 (15), however this mechanism requires confirmation through TNFα neutralization experiments.

An unusual observation that a subset of CD4^+^ T-cells in Mtb-infected humans and macaques retain IL-22 at the cell membrane instead of secreting it was reported by Zeng et al. who speculated that a membrane-bound IL-22 may enjoy longer half-life. The authors show that IL-22^+^CD4^+^ T cells reduce intra-macrophagic mycobacteria by direct cell-to-cell contact, however whether the anti-mycobacterial effect is indeed mediated by direct interaction of membrane-bound IL-22 on T cells with the IL-22R1 on macrophages remains to be corroborated with additional data (14).

**Figure 1:**
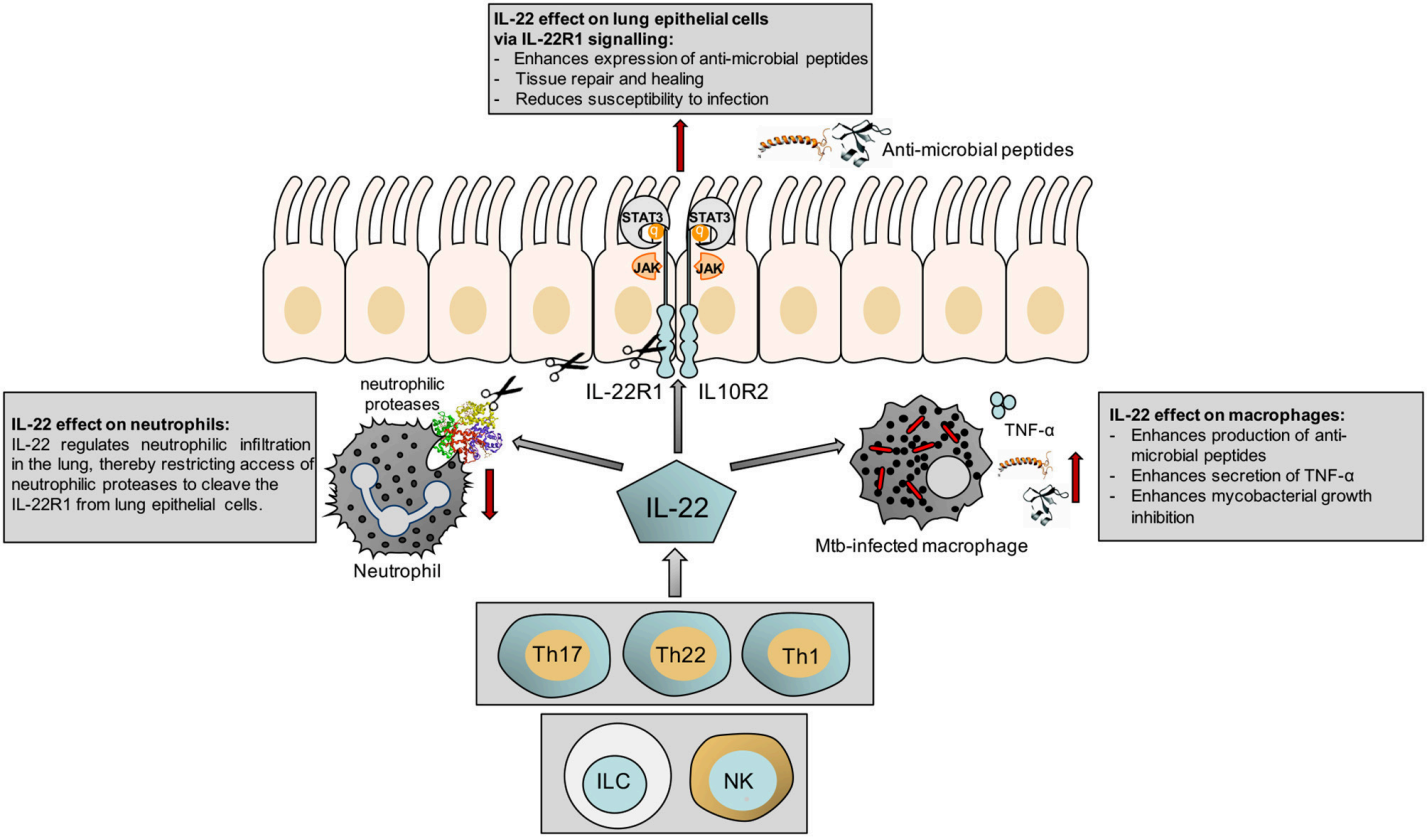
Schematic illustrating the effect of IL-22 on the epithelium, macrophages and neutrophils.

The responsiveness of macrophages to IL-22 has also been shown in a different context, where IL-22 modulates cholesterol efflux from macrophages (24). This may have implications for control of mycobacteria, which catabolize host sterols to sustain a persistent infection (25). A summary of our current knowledge of the actions of IL-22 is shown in Figure 1.

## The role of IL-22 in experimental animal models of lung infection

In an experimental murine model of *Streptococcus pneumoniae* rapid accumulation of IL-22 producing ILC3 in the lungs were observed and associated with protection from lethal infection (26). IL-22^(−/−)^ mice had greater streptococcal burden compared to wild-type mice and administration of rIL-22 reduced bacterial burden (27). Similarly, reduction of IL-22 production by depletion of ILCs in a *Pseudomonas aeruginosa* murine model induced lung injury was associated with reduced survival (28) pointing toward a host-protective role of IL-22 in both lung infection models. Interestingly, antibody-based neutralization of IL-22 led to increased neutrophilic infiltration and susceptibility to *P. aeruginosa* (29). This observation is consistent with the finding that IL-22 reduces expression of the neutrophil attracting chemokine CXCL8 from lung epithelial cells (17) and therefore a lack of IL-22 likely increases CXCL8, which in turn may drive the neutrophilic infiltration.

Neutrophilic proteases were previously shown to cleave the IL-22R1 on human bronchial epithelial cells and impair IL-22-dependent β-defensin expression, potentially contributing to pathogen replication (30). Administration of rIL-22 or neutralization of IL-22BP resulted in a decrease in lung damage and increased survival (29). Interestingly, *P. aeruginosa* has developed an immune-evasion strategy by secreting a serine protease which cleaves IL-22 resulting in its degradation, thereby weakening tissue repair and the anti-microbial defense (30). It will be interesting to investigate whether Mtb has acquired similar immune-evasion strategies and possesses proteases which cleave IL-22 and thus impair the IL-22R signaling pathway and host tissue repair. A murine model of *Haemophilus influenzae* infection further confirmed the beneficial effects of IL-22 observed in *P. aeruginosa* infected mice, where IL-22^(−/−)^ mice had increased bacterial burden and administration of exogenous IL-22 boosted bacterial clearance and limited lung tissue damage (31). IL-22 secretion by Th17 cells is crucial for control of the Gram-negative pulmonary pathogen *K. pneumoniae* and promotion of lung epithelial cell proliferation (16). IL-22 also reduces lung inflammation during influenza A virus infection and protect against secondary bacterial infection (22). In summary, there is evidence from various lung infection models that IL-22 plays a protective effect on host mucosal surfaces, whereas the effect of IL-22 on bacterial clearance appears to be pathogen-specific.

Mycobacterial infection models using IL-22^(−/−)^ mice have yielded conflicting results. IL-22^(−/−)^ mice infected with both high and low dose H37Rv had comparable pro-inflammatory cytokine profiles in the lung as wild-type C57BL/6 mice with exception of increased IL-6 and reduced MMP-9 and CXCL-10 (32). Recruitment of macrophages and granulocytes to the lung were similar between IL-22^(−/−)^ and wild-type mice and there were no significant differences in bacterial burden and survival. Similar to the studies in IL-22^(−/−)^ mice, administration of an IL-22 neutralizing antibody to wild-type mice 12 weeks post-infection did not compromise survival or alter bacterial burden (33). However, the timing of anti-IL22 administration may be crucial as the *P. aeruginosa* infection model suggests that elevated IL-22 concentrations prior to infection are important for conveying the protective effects (29). The Mtb infection studies in IL-22^(−/−)^ mice by Behrends and colleagues were carried out with H37Rv and different results were obtained when the knock out mice were infected with HN878 (15). Mtb HN878 infection induced IL-22 production *via* a TLR2 and IL-1β-dependent pathway and in this model IL-22 plays an important role in recruitment of myeloid cells to the lung (15). IL-22^(−/−)^ mice were more susceptible and exhibited higher bacterial burden during the chronic stage of HN878 infection 100 days post-infection, whereas no differences in susceptibility were observed during the acute phase 30 days post-infection. Therefore, these data suggest that susceptibility of IL-22^(−/−)^ mice to mycobacteria is largely driven by the infection stage (acute vs. chronic) and the mycobacterial strain (H37Rv, Erdman vs. HN878). Strain specific differences in eliciting an IL-22 response have also been shown in PBMCs from TB patients stimulated with cell wall extracts from Mtb HN878, which resulted in greater production of IL-22 compared to H37Rv cell wall extracts (15). In addition, as the timing of exogenous administration of IL-22 appears to be important from other infection models, further studies in IL-22^(−/−)^ mice with administration of IL-22 prior to Mtb infection are required. Such studies are however complicated by the short half-life of IL-22 and its off-target effects on mucosal tissues other than the lung.

In a non-human primate model, Mtb infection resulted in reduced IL-22 mRNA expression in peripheral blood but increased expression in the lymphocytes of the lungs, bronchial lymph nodes, and the spleen (34). These observations from primates are consistent with human studies where elevated IL-22 protein was found at the site of disease in human broncho-alveolar lavage fluid (BALF) (35–37) as well as increased percentages of IL-22^+^ CD4^+^ T cells in BALF compared to blood (38). Although elevation of IL-22 in primates was associated with severe TB, it is not clear whether IL-22 production is induced as a consequence of enhanced inflammation to counteract immunopathology or directly contributes to pathology itself. IL-22 producing T cells were also observed in BALF from Mtb-infected compared to un-infected primates and were visualized in TB granulomas by immunohistochemistry (39). Furthermore, IL-22 expression was also found in lung and lymph node granulomas of *Mycobacterium bovis* infected cattle (40). Interestingly, in this species IL-22 was shown to be one of the dominant surrogates of protection from bovine TB after *M. bovis Bacille-Calmette-Guerin* (BCG) vaccination (41). Whether IL-22 is a surrogate of protection from human TB remains to be established.

## IL-22 in human latent and active TB

In humans Mtb induces a distinct antigen specific IL-22^+^ CD4^+^ T cell population with central memory phenotype, which was first identified in antigen stimulated whole blood from mycobacteria exposed individuals (35). People with latent TB infection (LTBI), who have not progressed to active TB, have significantly higher frequencies of these Mtb specific IL-22 producing CD4^+^ cells compared to active TB patients (42), which is consistent with the increased frequencies in IFNγ-producing Th1 cells during LTBI vs. TB. It is likely that both the Th1 and Th22 cell populations in addition to Th17 cells contribute to protection from progression to TB. Furthermore, a single nucleotide polymorphism in the promoter of the IL-22 gene, which is associated with higher Mtb-antigen specific IL-22 production from PBMCs is over-represented in controls compared to TB patients suggesting that it is associated with reduced susceptibility to TB (43). Some studies report higher serum concentrations of IL-22 in individuals with LTBI compared to TB patients (44–46), whereas other studies do not show significant differences in circulating IL-22 concentrations (42). These different observations may be due to the ethnic background and Mtb strains prevalent in the respective study cohorts.

At the site of disease however, several studies consistently report increased concentrations of IL-22 in BALF from TB patients compared to controls and higher IL-22 concentrations at the site of disease vs. peripheral blood (35–37), which may be due to migration of antigen specific IL-22 producing T cells to the site of disease, the lung. In patients with TB pleurisy, IL-22, and IFNγ were also elevated in pleural fluid as were antigen-specific IL-22 producing CD4+ T cells (47). In patients with extra-pulmonary TB-associated pericardial and pleural effusions IL-22 concentrations correlated with MMP-9 expression (36). However, whether IL-22 contributes to immunopathology or is produced to counteract immunopathology was not established in this context, although MMP-9 production has been linked to improved epithelial barrier function in the gut (48) and it is possible that IL-22 and MMP-9 are induced in order to promote healing rather than being drivers of immunopathology. Successful TB treatment restores antigen-specific IL-22 responses by reducing the frequencies of CD19+CD1d+CD5+ regulatory B cell, which were shown to suppress IL-22 production (49).

In patients with *Mycobacterium avium complex (MAC)* infection, low IL-22 concentrations in BALF were associated with a neutrophil dominant inflammatory response, radiological severity and progression to pulmonary MAC disease, whereas individuals high IL-22 concentrations in BALF had greater percentages of lymphocytes and less disease severity (50). This finding is consistent with the observation that IL-22 regulates neutrophilic infiltration as shown in an animal model of lung infection (29). Collectively these data point toward an important role of IL-22 in mycobacterial infection and highlight the need to further define its role in progression from latent to active TB as well as in treatment outcomes.

## IL-22 in TB-diabetes co-morbidity

The threat of TB and diabetes (T2D) comorbidity to TB control programs is well recognized, but the underlying mechanisms contributing to increased susceptibility of T2D patients to TB and the increased risk of poor treatment outcomes in patients with TB-T2D comorbidity are poorly understood (51, 52).

T2D patients with LTBI have lower frequencies of Mtb-specific Th1, Th17, and Th2 responses compared to normo-glycemic individuals with LTBI. Once T2D patients develop TB they exhibit higher circulating concentrations of Th1 and Th17 cytokines compared to TB patients without T2D (52). Despite this increased production of Th1 and Th17 cytokines, which are important for protective immune responses to Mtb, TB-T2D patients are more likely to fail treatment and relapse after initial cure (53). Interestingly, IL-22 is the only cytokine found at lower concentrations in serum of TB-T2D patients compared to TB patients without co-morbidities (44, 46), but a causative link between low IL-22 serum concentrations and risk of poor treatment outcomes is far from established. Interestingly, Kumar et al. reported that T2D patients with LTBI had higher IL-22 serum concentrations compared to individuals with LTBI and no T2D (45). Although this appears puzzling, it is possible that latently infected T2D patients with high basal concentrations of IL-22 are less likely to progress to active disease.

A study based on a high fat diet mouse model of T2D showed that the induction of IL-22 from CD4^+^ cells is impaired in obese mice in response to challenge with the intestinal pathogen *Citrobacter rodentium*, making them more susceptible to infection. This defect was restricted to IL-22 producing T cells and IL-22 secretion by ILCs was not affected (54). Administration of rIL-22 not only improved mucosal host defense, but also many of the metabolic symptoms including hyperglycemia and insulin resistance in this and another murine T2D model (54, 55). This further raises the question whether IL-22 may be useful as adjunct host-directed therapy in the context of TB-T2D.

## Conclusions

IL-22 is a key regulator of immunity and inflammation at mucosal surfaces including the lung. Current evidence suggests that an optimal amount of this cytokine prior to infection can contribute to containment of bacteria and to protection from excessive tissue damage. The contribution of IL-22 and Mtb-specific IL-22^+^ T cells in protection from progression to TB in presence and absence of T2D co-morbidity in humans and the importance of this cytokine in TB treatment response requires further studies.

## Author contributions

KR, RS, and MC reviewed the current literature. KR and RS wrote the manuscript. All authors critically reviewed the manuscript and MC created Figure 1.

### Conflict of interest statement

The authors declare that the research was conducted in the absence of any commercial or financial relationships that could be construed as a potential conflict of interest. The reviewer WB and handling Editor declared their shared affiliation.
